# Mechanisms Associated with Quality Deterioration for Vacuum-Packaged Pork Jerky During Accelerated High-Temperature and High-Humidity Storage

**DOI:** 10.3390/foods15142565

**Published:** 2026-07-21

**Authors:** Yankun Fu, Changcheng Zhao, Xiaolin Liang, Rong Liu, Pengjie Wang, Shumin Wang

**Affiliations:** 1 Department of Nutrition and Health, China Agricultural University, Beijing 100193, China; hntyfyk2021@163.com (Y.F.); lynnloeng13@163.com (X.L.); liurong@cau.edu.cn (R.L.); 2 School of Life Science, Zhengzhou University, Zhengzhou 450001, China; zhaocc91@163.com

**Keywords:** pork jerky, lipid oxidation, protein oxidation, Maillard reaction, quality deterioration

## Abstract

Pork jerky is vulnerable to quality deterioration during distribution, particularly under hot and humid conditions. This study investigated the deterioration pattern and associated mechanisms for vacuum-packaged pork jerky sealed in aluminum foil pouches during accelerated high-temperature and high-humidity storage (65 °C and relative humidity of 85%). The sensory quality, physicochemical properties, oxidation-related changes, non-enzymatic browning, and protein digestibility were evaluated, and multivariate analyses were performed to characterize the deterioration process. Sensory acceptability declined markedly, and the rejection rate reached 50% on day 14, indicating the shelf-life endpoint under the test conditions. Deterioration in quality was mainly characterized by color darkening, texture hardening, water redistribution, and microstructural disruption. Lipid oxidation, protein oxidation, myoglobin oxidation, and non-enzymatic browning progressively intensified during storage and were associated with the observed color deterioration and sensory decline. Microbiological indicators remained below the detection limit throughout storage, suggesting that quality deterioration was primarily associated with chemical rather than microbial changes. Multivariate analyses further suggested that lipid oxidation, protein oxidation, myoglobin oxidation, and non-enzymatic browning were key processes potentially associated with quality loss. These findings provide a basis for shelf-life evaluation and quality control for pork jerky under extreme storage conditions.

## 1. Introduction

Pork jerky is one of the best-known traditional dried meat products consumed in China. It is highly popular because of its high energy density, rich flavor, convenience for storage and transportation, and ease of consumption, and has become an important type of meat snack in Asia and worldwide [1,2]. In recent years, global warming has led to significant increases in the frequency and duration of high-temperature and high-humidity weather. In many regions around the world, high summer temperatures above 40 °C have become common. At the same time, due to the growing consumer demand for snack foods and rapid development of e-commerce logistics systems, the sales range of pork jerky has continued to expand, and export regions have increasingly extended to tropical and subtropical areas with high-temperature and high-humidity conditions. In addition, pork jerky manufacturers are mainly concentrated in the southeastern coastal areas of China, where the ambient temperature and relative humidity (RH) are relatively high throughout the year. Therefore, during production, storage, transportation, and sales, pork jerky products are increasingly affected by challenging high-temperature and high-humidity environments.

However, high-temperature environments may significantly increase the risk of pork jerky quality deterioration, and studies have shown that the storage temperature is a key factor affecting the stability of meat product quality. Wazir et al. found that as the storage temperature increased, quality deterioration was significantly aggravated for low-moisture meat products [3]. Domínguez et al. also showed that high temperature can accelerate oxidative chain reactions, thereby promoting rancidity, discoloration, and flavor deterioration in meat products [4]. In recent years, some studies have reported changes in the quality of dried meat products during storage. Color, flavor, and texture deterioration have been observed [3,5,6], and oxidation and non-enzymatic browning may be the main causes of quality decline in meat products [7,8,9]. However, most previous studies were conducted under refrigerated or room-temperature storage conditions, whereas few studies investigated quality deterioration phenomena and the dominant mechanisms involved for pork jerky under high-temperature and high-humidity conditions. Different food reactions have different apparent activation energies, and thus the kinetic processes involved in chemical reactions such as lipid oxidation, protein oxidation, pigment degradation, and the Maillard reaction may change significantly under high-temperature and high-humidity environments, and the dominant factors and mechanisms associated with quality deterioration may be essentially different from those under room-temperature conditions [10]. Therefore, it might not be appropriate to directly extrapolate research findings obtained under room-temperature conditions to high-temperature and high-humidity environments.

Accelerated shelf-life testing has been widely used in food shelf-life evaluations. The quality deterioration process is accelerated by increasing the storage temperature or humidity, and thus the deterioration patterns and key mechanisms that affect products can be assessed within a shorter time [11]. Previous studies have confirmed that during hot summer weather, the internal temperatures of transport vehicles, containers, and storage facilities can be significantly higher than the ambient temperature, and may even approach or exceed 60 °C in local areas [12,13]. Therefore, constructing a high-temperature and high-humidity accelerated storage model is highly significant for evaluating the stability of pork jerky quality under extreme environments, and analyzing the dominant associated deterioration mechanisms. Consequently, the present study investigated vacuum-packaged pork jerky, and according to the principle of accelerated shelf-life testing, the quality deterioration process was accelerated within an acceptable experimental period, and accelerated storage experiments were carried out at 65 °C and 85% RH. The changes in quality-related indicators for pork jerky during storage were systematically characterized, and the mechanisms involved were comprehensively analyzed. Correlation analysis and multivariate statistical analysis were combined to explore the dominant factors related to quality deterioration in order to provide a theoretical basis for evaluating quality stability, shelf-life management, and subsequent stability optimization for pork jerky under high-temperature and high-humidity environments.

## 2. Materials and Methods

### 2.1. Materials

Pork jerky was obtained from Jiangsu Shuangyu Food Co., Ltd. (Jingjiang, China). According to the manufacturer, the product was prepared primarily from pork hind leg meat and contained sucrose, egg, fish sauce, spices, monosodium glutamate, sodium erythorbate, and disodium 5′-ribonucleotide as ingredients. The pork jerky samples were vacuum-packaged in three-layer high-barrier aluminum foil pouches (PET/Al/PE structure). The pouch dimensions were 7 cm × 12 cm. The oxygen transmission rate and water vapor transmission rate were 0.04 ± 0.01 cm^3^ m^−2^ 24 h^−1^ 0.1 MPa and 0.00050 ± 0.00025 g m^−2^ 24 h^−1^, respectively. The puncture strength of the packaging material was 8.48 ± 0.12 N. The initial water activity in the pork jerky was 0.57 ± 0.001. The samples were stored at 65 °C and 85% RH for 14 days and collected at 0, 2, 4, 6, 8, 10, 12, and 14 days for subsequent analyses. All samples used in this study were obtained from the same production batch to minimize batch-to-batch variation. All chemicals and reagents were analytical grade. Ferrous chloride, simulated gastric fluid, simulated intestinal fluid, o-phthalaldehyde (OPA), potassium thiocyanate, and pepsin were purchased from Beijing Solarbio Science & Technology Co., Ltd. (Beijing, China). Trypsin was purchased from Shanghai yuanye Bio-Technology Co., Ltd. (Shanghai, China). Total sugar content assay kits and protein carbonyl content assay kits were purchased from Boxbio Biotech Co., Ltd. (Beijing, China). All other reagents were purchased from Sinopharm Chemical Reagent Co., Ltd. (Shanghai, China).

### 2.2. Sensory Quality

#### 2.2.1. Sensory Evaluation

Sensory evaluations were conducted based on sensory quality assessments by a trained panel to monitor the quality of pork jerky during storage. A panel was recruited consisting of 10 trained food professionals (aged 22–35 years, mixed genders) with prior experience in meat product evaluation and no reported sensory impairments. Panelists underwent three training sessions each for 1 h to familiarize themselves with pork jerky attributes, scoring criteria, and reference standards for color, aroma, taste, texture, appearance, and overall acceptability.

Samples (2–3 g) were served at room temperature (25 °C) in individual sensory booths under controlled lighting, coded with random three-digit numbers, and presented in randomized order. Each sample was evaluated once at each storage time point by each panelist. Panelists evaluated color, aroma, taste, texture, and appearance using a 9-point sensory quality scale, where scores of 1–3, 4–6, and 7–9 represented poor, moderate, and good quality, respectively. Overall acceptability was evaluated using a 9-point hedonic scale, where scores of 0–1, 2–3, 4–5, 6–7, and 8–9 corresponded to “unacceptable,” “dislike,” “neither like nor dislike,” “like,” and “like very much,” respectively. Panelists were instructed to cleanse their palates with water between samples. In addition to sensory scoring, panelists were asked to indicate whether each sample was acceptable or unacceptable. The sensory rejection rate was calculated as the percentage of panelists who considered the sample unacceptable. The sensory shelf-life endpoint was defined as the storage time at which the rejection rate reached 50% [14,15].

All samples were food-grade and posed no physical or psychological risk to the panelists. According to relevant Chinese regulations, this type of sensory evaluation does not require formal ethics review. Informed consent was obtained from all participants prior to evaluation.

#### 2.2.2. Macro Images

Macro images of the samples were captured using a digital camera (EOS 750D, Canon Inc., Tokyo, Japan) equipped with a macro lens. During image acquisition, the camera was fixed in position and uniform illumination was applied. The white balance in the images was standardized using Adobe Photoshop software 2024 (Adobe Inc., San Jose, CA, USA).

### 2.3. Physicochemical Analyses

#### 2.3.1. Color Measurements

The colors of the pork jerky samples were determined using a colorimeter (CR-400, Konica Minolta Sensing Inc., Tokyo, Japan). The colorimeter was calibrated with standard porcelain white discs before tests. Prior to color measurement, samples were removed from the storage chamber and allowed to equilibrate at room temperature. Color measurements were then performed under ambient laboratory conditions. Subsequently, color measurements were obtained for each sample, and the average values of L*, a*, and b* were recorded. To improve reliability, measurements were performed for at least three randomly selected samples and the average values were used for the analysis. The Δ*E* values were determined for the pork jerky samples using the following formula:

(1) $$\Delta E\;=\;\sqrt{(L^{*}-\;{L_{0}}^{*}{)}^{2}+(a^{*}-{a_{0}}^{*}{)}^{2}+(b^{*}-{b_{0}}^{*}{)}^{2}}$$

where L*, a*, and b* are the color difference values measured for the samples, and *L*_0_*, *a*_0_*, and *b*_0_* are the color difference values for the initial samples.

#### 2.3.2. Texture Properties

The texture properties were determined for pork jerky samples using a TA.XT plus texture analyzer (Stable Micro Systems Ltd., Godalming, UK) according to the method described by Han et al. [16]. Hardness and shear force measurements were obtained using intact pork jerky slices without further cutting or reshaping. The pork jerky slices had approximate dimensions of 3 cm × 7 cm. Prior to texture analysis, samples were removed from the storage chamber and allowed to equilibrate at room temperature to minimize the effects of temperature and moisture differences on texture measurements. Hardness was measured by compression testing using a P/36 cylindrical probe under the following conditions: pre-test speed, 1.0 mm s^−1^; test speed, 1.0 mm s^−1^; post-test speed, 5.0 mm s^−1^; trigger force, 5 g; and a compression ratio of 40% of the original sample thickness. The maximum force obtained during compression was defined as the hardness. The shear force was determined using a shear blade (A/MORS). The sample was placed at the center of the testing platform and sheared perpendicular to the fiber direction at a crosshead speed of 180 mm min^−1^, with an initial force of 0.5 N, return distance of 8 mm, and load cell capacity of 500 N. The maximum force required to rupture the sample was recorded as the shear force. Six parallel measurements were performed for each group of samples.

#### 2.3.3. Microbial Analysis

Total viable counts (TVCs) were determined according to the Chinese National Food Safety Standard GB 4789.2-2022 with slight modifications [17]. Briefly, 10 g of each pork jerky sample was weighed aseptically and homogenized with 90 mL of sterile 0.85% saline for 1 min. Serial 10-fold dilutions were prepared and 1 mL aliquots of appropriate dilutions were spread on plate count agar. The plates were incubated at 36 ± 1 °C for 48 h, and visible colonies were counted. TVC results were expressed as colony-forming units (CFU) g^−1^ sample.

Yeast and mold counts were determined according to the Chinese National Food Safety Standard GB 4789.15-2016 [18]. Briefly, 25 g of pork jerky was aseptically transferred into a sterile homogenization bag containing 225 mL of sterile physiological saline and homogenized for 1–2 min. Appropriate tenfold dilutions were prepared using sterile physiological saline. Subsequently, 1 mL of the appropriate dilution was transferred into a sterile Petri dish, before adding approximately 15–20 mL of molten Rose Bengal Agar cooled to 46 ± 1 °C. The medium and sample were mixed thoroughly and allowed to solidify. The plates were incubated at 28 ± 1 °C for 5 days, before counting colonies of yeasts and molds. The results were expressed as CFU g^−1^ sample.

*Bacillus cereus* counts were determined according to the Chinese National Food Safety Standard GB 4789.14-2014 [19]. Briefly, 25 g of pork jerky was aseptically homogenized with 225 mL of sterile physiological saline, before preparing appropriate tenfold dilutions. Aliquots of the appropriate dilutions were inoculated onto Mannitol Egg Yolk Polymyxin (MYP) agar and incubated at 30 °C for 24 h. Typical colonies were counted and the results were expressed as CFU g^−1^ sample.

#### 2.3.4. Water Status

Moisture contents were determined using the direct drying method, according to Chinese National Food Safety Standard GB 5009.3-2016 [20]. Briefly, 2 g of finely minced pork jerky was accurately weighed in a pre-dried and pre-weighed moisture dish. The sample was dried until a constant weight was reached in a hot-air oven at 105 °C (i.e., the difference between two consecutive weighings was <2 mg). After cooling to room temperature in a desiccator, the dish was weighed again. The moisture content was calculated based on the weight loss after drying and expressed as g 100 g^−1^ sample:

(2) $$X=\frac{m_{1}-m_{2}}{m_{1}-m_{3}}\times 100$$

where *m*_1_ is the mass of the weighing bottle and sample before drying (g), *m*_2_ is the mass of the weighing bottle and sample after drying (g), and *m*_3_ is the mass of the empty weighing bottle (g).

The water distributions and proportions in the samples were determined using a low-field nuclear magnetic resonance (LF-NMR) instrument (NMI20-060V-I) according to the method described by Shao et al. [21], with slight modifications. Approximately 2 g of undried pork jerky was placed in an NMR tube with a diameter of 25 mm for measurement. Prior to analysis, the instrument was calibrated using rapeseed samples according to the manufacturer’s instructions. After normalizing the raw data, the transverse relaxation time (T_2_) was measured using the Carr–Purcell–Meiboom–Gill pulse sequence. The main operating parameters were as follows: radiofrequency delay, 0.02 ms; pre-scan gain, 2; waiting time, 500 ms; and relaxation time, 100,000 ms. Inversion analysis was performed using CONTIN software 3.0 (Bruker BioSpin GmbH, Rheinstetten, Germany).

The Water activity (aw) was determined according to the Chinese National Food Safety Standard GB 5009.238-2016 [22]. Briefly, 2 g of finely minced pork jerky was placed into the sample cup and allowed to equilibrate at room temperature. The water activity was then measured using a water activity meter until a stable reading was obtained. The results were expressed as aw.

#### 2.3.5. Protein and Fat Contents

Protein contents were determined using the Kjeldahl method, according to Chinese National Food Safety Standard GB 5009.5-2025 [23]. Briefly, 1 g of minced pork jerky was digested with concentrated sulfuric acid in the presence of a catalyst until a clear solution was obtained. The digested solution was neutralized and distilled with NaOH, and the released ammonia was trapped in a boric acid solution and titrated with standard hydrochloric acid. The protein content was calculated by multiplying the nitrogen content by a conversion factor of 6.25 and expressed as g 100 g^−1^ sample.

Fat contents were determined using the Soxhlet extraction method according to Chinese National Food Safety Standard GB 5009.6-2016 [24]. Briefly, 2–3 g of minced pork jerky was placed in a cellulose extraction thimble and extracted with petroleum ether for 6 h. The solvent was removed by rotary evaporation, and the residual fat was dried to constant weight at 105 °C. The fat content was expressed as g 100 g^−1^ sample.

#### 2.3.6. pH

The pH was determined according to the method described by Lim et al. with slight modifications [25]. Briefly, 3 g of pork jerky was cut into small pieces and homogenized with 27 mL of distilled water (1:9, *w*/*v*) according to the extraction ratio reported by Lim et al. [25]. The homogenate was centrifuged at 4000× *g* for 10 min and filtered. The pH of the filtrate was measured using a pH meter. Each sample was analyzed in triplicate.

#### 2.3.7. Scanning Electron Microscopy (SEM) Observations

Samples were collected from the central part of the pork jerky and cut into small pieces measuring approximately 5 mm × 5 mm × 2 mm. The samples were fixed in 2.5% glutaraldehyde at 4 °C for 24 h, before dehydration using a graded ethanol series, drying, and gold sputtering. The microstructures of the samples were observed by using SEM according to the method described by Li et al. with slight modifications [26]. Samples were examined at magnifications of 50×, 100×, and 200×, and the accelerating voltage was set at 3 kV. For each treatment group, multiple fields of view were examined, and representative images showing the typical microstructural characteristics of the samples were selected for presentation.

### 2.4. Lipid Oxidation

#### 2.4.1. Peroxide Value (PV)

PVs were determined using the ferric thiocyanate colorimetric method according to Hultin et al. with slight modifications [27]. Briefly, lipids were extracted from 15 g of pork jerky using petroleum ether at a sample-to-solvent ratio of 1:2 (*w*/*v*). Extraction was conducted for a fixed period of time and completed under limited light conditions in order to minimize the possibility of lipid oxidation during the sample preparation process. An aliquot of 0.25 g of the extracted lipid was dissolved in chloroform–methanol (2:1, *v*/*v*), before adding NaCl solution to induce phase separation. After centrifugation, the lower phase was collected and diluted, and potassium thiocyanate and ferrous chloride solutions were added sequentially for color development. After incubating at room temperature in the dark for 5 min, the absorbance was measured at 500 nm. The results were calculated from an iron ion standard curve and expressed as mEq kg^−1^ lipid.

#### 2.4.2. Thiobarbituric Acid Reactive Substances (TBARSs)

TBARS values were determined according to the method described by Aung et al. with slight modifications [28]. Briefly, 1.5 g of minced pork jerky was homogenized with 10 mL of trichloroacetic acid solution (0.075 g mL^−1^) containing 0.1% EDTA at 15,000× *g* for 30 s, followed by centrifugation at 4000× *g* for 10 min and filtration. Next, 5 mL of the filtrate was mixed with 5 mL of 0.02 mol L^−1^ thiobarbituric acid solution. A blank was prepared using 5 mL of trichloroacetic acid solution instead of the sample extract for comparison. The mixtures were heated in a water bath at 90 °C for 30 min, before cooling to room temperature and measuring the absorbance at 532 nm. The TBARS value was calculated from the standard curve for malondialdehyde (MDA) and expressed as mg MDA kg^−1^:

(3) $$X=\frac{cV}{m}$$

where *X* represents the MDA content of the sample (mg MDA kg^−1^), *c* is the MDA concentration in the sample (μg mL^–1^), *V* is the final volume of the sample solution (mL), and *m* is the mass of the sample (g).

### 2.5. Protein Oxidation

#### 2.5.1. Detection of Myoglobin Forms

Myoglobin forms were determined according to the method described by Chen et al. with slight modifications [29]. Briefly, 5 g of each chopped pork jerky sample was homogenized with 25 mL of 0.04 mol L^−1^ phosphate buffer (pH 6.8) in a 50 mL centrifuge tube. The homogenate was held at 4 °C for 1 h and then centrifuged at 10,000× *g* and 4 °C for 25 min. The supernatant was filtered through a 0.45 μm membrane filter. The absorbance of the filtrate was measured at 525, 545, 565, and 572 nm using a microplate reader. The oxymyoglobin (OMb), deoxymyoglobin (DMb), and metmyoglobin (MMb) contents were calculated using the following equations:

(4) $$\mathrm{DMb}(\%)\;=\;(0.369\;\times \;R_{1}\;+\;1.140\;\times \;R_{2}-\;0.941\;\times \;R_{3}\;+\;0.015)\;\times \;100$$

(5) $$\mathrm{OMb}(\%)=(0.882\;\times \;R_{1}-1.267\;\times \;R_{2}+0.809\;\times \;R_{3}-0.361)\;\times \;100$$

(6) $$\mathrm{MMb}(\%)=(-2.541\;\times \;R_{1}+0.777\times R_{2}+0.800\;\times {\;R}_{3}+1.098)\;\times \;100$$

where $\begin{array}{c} R_{1}=A_{572\mathrm{nm}}/A_{525\mathrm{nm}},\; \end{array}R_{2}=A_{565\mathrm{nm}}/A_{525\mathrm{nm}}{,\;\mathrm{and}\;R}_{3}=A_{545\mathrm{nm}}/A_{525\mathrm{nm}},$ respectively.

#### 2.5.2. Total Volatile Basic Nitrogen (TVB-N)

The TVB-N contents were determined according to Chinese National Food Safety Standard GB 5009.228-2016 [30] using the semimicro Kjeldahl diffusion method. Briefly, 5.00 g of minced pork jerky was extracted with distilled water, and the volatile basic nitrogen compounds were released under alkaline conditions and absorbed by boric acid. The absorbed ammonia was titrated with standard hydrochloric acid solution, and the TVB-N content was calculated and expressed as mg 100 g^−1^ sample.

#### 2.5.3. Total Sulfhydryl Content

The sulfhydryl contents were determined according to Zhang et al. with slight modifications [31]. Briefly, 0.5 mL of protein solution (2 mg mL^−1^) was mixed with 2.5 mL of Tris–Gly buffer containing 8 M urea, before adding 0.02 mL 5,5′-dithiobis (2-nitrobenzoic acid) (DTNB) solution (4 mg mL^−1^). The mixture was incubated at 25 °C for 30 min and the absorbance was measured at 412 nm. The total sulfhydryl content was calculated using the following equation:

(7) $$Total\;sulfhydryl\;content\;(\mu mol/g)\;=\frac{73.53\;\times \;A412\;\times \;D}{C_{pro}}$$

where *D* is the dilution factor and *C_pro_* is the protein concentration.

#### 2.5.4. Carbonyl Content

Protein carbonyl contents were measured using the corresponding assay kit from Boxbio (Beijing, China) according to the manufacturer’s instructions. Briefly, 0.2 g of pork jerky was homogenized in extraction buffer, before the homogenate was centrifuged to collect the supernatant. The supernatant was then reacted sequentially with the kit reagents according to the manufacturer’s instructions. After the incubation and centrifugation steps, the absorbance of the final solution was measured at 370 nm using an ultraviolet–visible spectrophotometer. The protein carbonyl content was calculated based on the difference in absorbance between the sample and control, and expressed as μmol g^−1^ protein.

#### 2.5.5. Schiff Base Content

The Schiff base contents were determined according to the method described by Estévez et al. with slight modifications [32]. Briefly, the extracted soluble protein was dissolved in 20 mM phosphate-buffered saline to a final concentration of 1 mg mL^−1^. The excitation wavelength was set at 350 nm and the emission spectra were recorded from 400 to 500 nm, where both the excitation and emission slit widths were set at 10 nm. The scanning rate was set to 600 nm min^−1^. The results were expressed as the fluorescence intensity at 460 nm.

### 2.6. Maillard Reaction

To evaluate the progression of non-enzymatic browning during storage, several indicators associated with different stages of the Maillard reaction were determined. Reducing sugars and free amino acids were analyzed as major reaction substrates, and the total sugar content was also determined to monitor the overall consumption and transformation of sugar components during storage. Schiff base formation was used to reflect the early stage of carbonyl–amino condensation and the browning degree (absorbance at 420 nm (A420)) was applied as an indicator of advanced browning product formation.

#### 2.6.1. Total Sugar Content

Total sugar contents were determined using a Total Sugar Content Assay Kit (Boxbio, Beijing, China) according to the manufacturer’s instructions. Briefly, 0.1 g of pork jerky was homogenized with extraction buffer and hydrolyzed in a boiling water bath for 30 min. After cooling, the extract was centrifuged and the supernatant was collected for analysis. The supernatant was reacted with 3,5-dinitrosalicylic acid (DNS) color reagent in a boiling water bath for 10 min, and then cooled to room temperature. The absorbance was measured at 540 nm using a spectrophotometer. The total sugar content was calculated from a standard curve for glucose and expressed as mg g^−1^ sample.

#### 2.6.2. Reducing Sugar Content

The reducing sugar contents were determined using the DNS method according to Yoo et al. with slight modifications [33]. Briefly, each pork jerky sample was chopped and homogenized, and 0.50 g of the homogenate was transferred to a 50 mL centrifuge tube. Next, 20 mL of 1% oxalic acid solution was added, and the mixture was extracted in an 80 °C water bath for 30 min. After cooling, the extract was diluted to 50 mL with 1% oxalic acid solution, centrifuged at 4000× *g* for 10 min, and filtered. Subsequently, 1 mL of the filtrate was mixed with 1 mL of DNS reagent and heated in a boiling water bath for 5 min. The reaction mixture was immediately cooled to room temperature and diluted to 10 mL using distilled water. The absorbance was measured at 540 nm, and the reducing sugar content was calculated using a standard curve for glucose. The measured results were expressed as percentages (%) and used for subsequent analyses.

#### 2.6.3. Free Amino Acid Content

The free amino acid contents were determined using the OPA derivatization method according to Nielsen et al. with slight modifications [34]. Briefly, 5.00 g of each pork jerky sample was mixed with 20 mL of 5% (*w*/*v*) trichloroacetic acid (TCA) solution, vortexed for 1 min, and kept at 4 °C for 30 min. The mixture was then centrifuged at 4000× *g* for 15 min, and the supernatant was collected and made up to 50 mL with 5% TCA solution. After filtration through a 0.45 μm membrane, the filtrate was used for analysis and diluted as appropriate when necessary. Next, 1.0 mL of the filtrate was mixed with 3.0 mL of OPA reagent and reacted in the dark at 25 °C for 2 min. The absorbance was measured at 340 nm. The free amino acid content was calculated from the standard curve and expressed as mg 100 g^−1^, according to the following equation:

(8) $$X=\frac{C\times V\times n}{m\times 1000}\times 100$$

where *X* is the free amino acid content of the sample (mg 100 g^−1^), *C* is the concentration of the target compound obtained from the standard curve, *V* is the final volume of the extract (50 mL), *n* is the dilution factor, and m is the sample mass (g).

#### 2.6.4. Browning Degree

The browning degree was determined according to the method described by Zhang et al. with slight modifications [35]. Briefly, 5 g of each ground pork jerky sample was transferred to a 50 mL centrifuge tube and homogenized with 10 mL of cold deionized water at 15,000× *g* for 1 min. Subsequently, 10 mL of cold 20% TCA solution was added and mixed thoroughly. The mixture was centrifuged at 5000× *g* and 4 °C for 10 min. The supernatant was filtered through a 0.22 μm membrane filter, and the filtrate was collected for further analysis. The absorbance of the filtrate was measured at 420 nm using a microplate reader, and the results were used to indicate the browning degree of the sample.

### 2.7. Protein Digestion Characteristics

Protein digestion properties were evaluated using a simulated in vitro gastrointestinal digestion model according to Hu et al. with slight modifications [36]. Briefly, 5.0 g of each sample was mixed with 6 mL of simulated gastric fluid and digested at 37 °C for 2 h with pepsin at a final activity of 2000 U mL^−1^ and pH 2.0. Next, 12 mL of simulated intestinal fluid was added to obtain final concentrations of 20 mM bile salts and 2000 U mL^−1^ pancreatin. The pH was maintained at 7.0 with 0.2 M NaOH, and digestion was continued at 37 °C for 2 h. All in vitro digestion experiments were performed in triplicate using independently prepared samples.

After digestion, the digesta were treated with ethanol, centrifuged, dried, and analyzed to determine the protein content using the Kjeldahl method in order to calculate protein digestibility [37].

The degree of hydrolysis (DH) was determined using the OPA method. After centrifuging the digesta, the supernatant was mixed with OPA reagent and reacted for 2 min, and the absorbance was measured at 340 nm. DH was calculated from the standard curve according to the method described by Nielsen et al. with minor modifications [34].

### 2.8. Data and Statistical Analysis

At each storage time point, three independent vacuum-packaged pork jerky samples were randomly selected as biological replicates (*n* = 3). Samples from each package were analyzed separately. All physicochemical analyses were performed in triplicate for each biological replicate, and the average value was used for statistical analysis. Results were expressed as the mean ± standard error of the mean based on three biological replicates. Statistical analyses were performed using GraphPad Prism (version 10.6.0; GraphPad Software, Boston, MA, USA). Differences among multiple groups were determined using one-way analysis of variance after assessing normality with the Shapiro–Wilk test and evaluating homogeneity of variance with the Brown–Forsythe test. Data that met the assumption of homogeneity of variance using the Brown–Forsythe test (*p* > 0.05) were analyzed with Tukey’s honestly significant difference test for post hoc multiple comparisons. Data that did not meet the assumption of homogeneity of variance (*p* < 0.05) were analyzed for post hoc multiple comparisons with Dunnett’s T3 test, which does not assume equal variances. The statistical test used for each data set was determined according to the homogeneity of variance assessment. Differences were considered statistically significant at *p* < 0.05. Multivariate statistical analyses were conducted using standardized data. Principal component analysis, Spearman’s correlation coefficients, and hierarchical cluster analysis were applied to evaluate the overall deterioration pattern and relationships among quality indicators during storage. Data were visualized using Origin 2026 software (OriginLab Corporation, Northampton, MA, USA).

## 3. Results and Discussion

### 3.1. Sensory Evaluation

**Figure 1:**
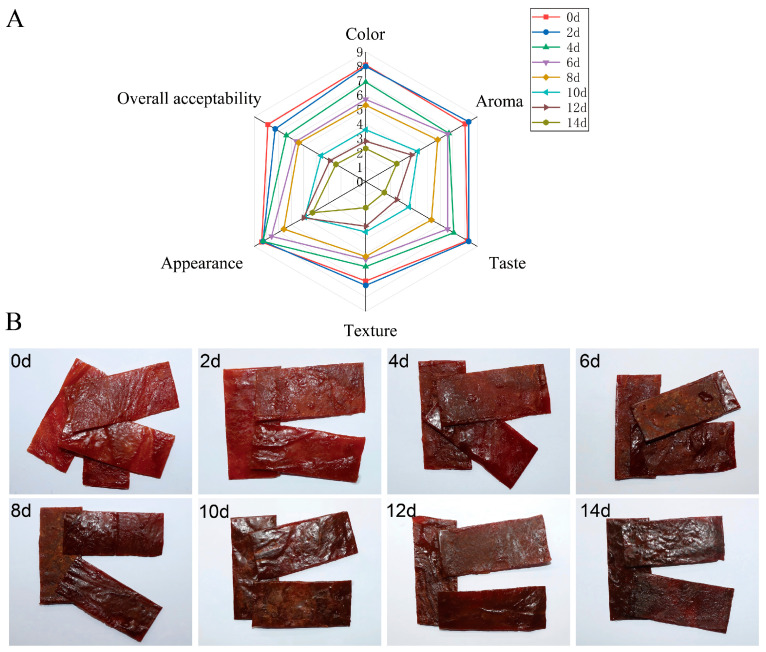
Changes in sensory scores (**A**) and macroscopic images (**B**) for pork jerky during storage.

Figure 1A presents a radar plot of the sensory scores for pork jerky during storage. The sensory scores decreased continuously during storage at 65 °C and 85% RH, with the most pronounced decline in color, followed by that in taste. As shown in Table 1, sensory rejection began on day 8 and the rejection rate reached 50% on day 14. According to the predefined sensory shelf-life criterion based on a 50% rejection threshold [38], the sensory shelf-life of pork jerky under the test storage conditions was determined as 14 days. The overall acceptability score decreased significantly from 7.90 ± 0.30 on day 0 to 2.40 ± 0.20 on day 14, indicating severe deterioration of pork jerky quality during the 14-day storage period. Detailed sensory scores for color, aroma, taste, texture, and appearance are provided in Supplementary Table S1. In addition, the macroscopic observations (Figure 1B) showed that the surface color of pork jerky gradually changed from reddish-brown to dark brown as the storage period progressed, which was accompanied by increasingly pronounced local darkening and color heterogeneity. This visual deterioration was consistent with the continuous decreases in the color, appearance, and texture scores shown in the radar plot. Similar declines in sensory quality have been reported for jerky and other dried meat products during storage [39,40]. Overall, the sensory evaluation results indicated that despite the use of vacuum packaging and high-barrier aluminum foil pouches, the pork jerky underwent rapid and multidimensional quality deterioration under the extreme storage conditions of 65 °C and 85% RH.

### 3.2. Physicochemical Analyses

#### 3.2.1. Changes in Color

**Figure 2:**
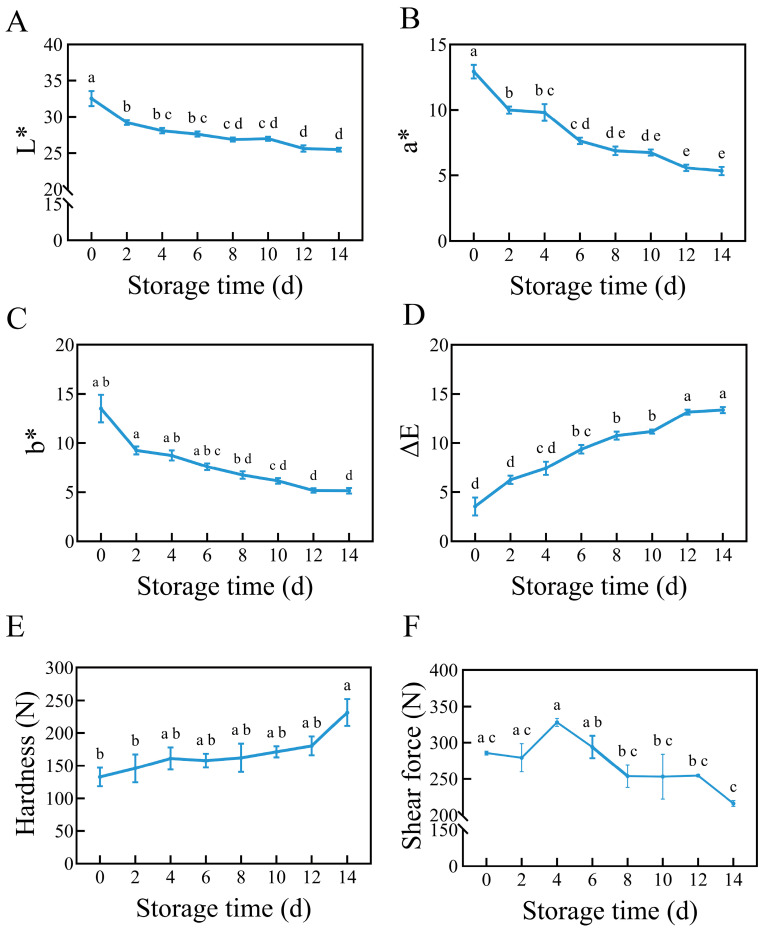
Changes in color and texture of pork jerky during storage. (**A**) L*; (**B**) a*; (**C**) b*; (**D**) ΔE; (**E**) hardness; and (**F**) shear force. Values represent the mean ± standard error (*n* = 3). Different lowercase letters indicate significant differences among storage times (*p* < 0.05).

As shown in Figure 2B, the a* value decreased markedly as the storage time increased, indicating continuous loss of redness, which is one of the most prominent features of color deterioration in pork jerky. A decrease in redness generally reflects the reduced stability of the meat pigment system, particularly enhanced myoglobin oxidation and the gradual conversion of OMb or other relatively stable red pigments into brown pigments, such as MMb, resulting in a color transition from bright red to dark brown [8]. In addition, the L* (Figure 2A) and b* (Figure 2C) values for pork jerky generally tended to decrease during storage, suggesting not only that pork jerky lost redness under high-temperature and high-humidity storage conditions but also that comprehensive color deterioration occurred. The samples gradually changed from relatively bright reddish-yellow to a darker and more brownish color, which was consistent with the macroscopic observations shown in Figure 1B. As shown in Figure 2D, the ΔE value gradually increased during prolonged storage, with more pronounced changes observed in the later stages, indicating that the overall color deviation from the initial state progressively increased, and the color deterioration became more severe in the late storage stage [41]. These color changes may have been associated with pigment oxidation and the accumulation of browning products during storage [8]. Similar decreases in color stability have been reported in jerky-type meat products during storage, where discoloration was accompanied by reductions in the quality of appearance and consumer acceptability [5,42].

#### 3.2.2. Changes in Texture

As shown in Figure 2E,F, the hardness generally tended to decrease initially but then increased with prolonged storage. A transient decrease was observed in the early stage of storage on day 2, followed by a continuous increase and then maintenance at a relatively high level. These findings suggest that pork jerky may have undergone a transition from early structural relaxation to subsequent tissue densification and hardening. Lian et al. reported that changes in the distribution of water can affect the microstructure and texture of meat products [43]. Similarly, Choi et al. found that jerky-type meat products are prone to increases in hardness and chewiness during storage, mainly due to protein denaturation, oxidative aggregation, and changes in the water status [44].

The hardness and shear force characterize different mechanical properties, where hardness reflects the resistance to compression and shear force indicates the resistance to cutting and fracture. In contrast to the hardness, the shear force increased initially and then decreased during storage. The simultaneous increase in hardness and decrease in shear force suggests that the pork jerky became harder but also more brittle. Protein oxidation-induced cross-linking initially increased the matrix rigidity [45], whereas continued oxidative damage and microstructural collapse, as shown by SEM analysis, reduced the integrity and continuity of the muscle fiber network [40]. Consequently, the samples became more susceptible to fracture during shearing, resulting in lower shear force values despite increased hardness. These findings suggest that the pork jerky gradually transformed from a relatively compact and tough structure into a hard but brittle structure during high-temperature and high-humidity storage.

#### 3.2.3. Changes in Microbial Abundance

As shown in Table 2, throughout the storage period, the TVC, yeast, mold, and *Bacillus cereus* counts were all below 10 CFU g^−1^, and thus within the limit range stipulated by the International Organization for Standardization (ISO), indicating that the monitored microorganisms did not undergo detectable proliferation under the experimental conditions. These observations may be attributed to the combined effects of the accelerated storage conditions (65 °C), low water activity of pork jerky, and vacuum packaging, which together limited microbial survival and proliferation under the test conditions [2,46]. These results suggest that microbial growth was not the dominant deterioration pathway under the accelerated storage conditions employed in this study.

#### 3.2.4. Changes in Water Status

As shown in Table 3, the moisture content of pork jerky gradually increased during storage, indicating that moisture uptake occurred under the high-temperature and high-humidity storage conditions despite vacuum packaging, possibly because these conditions weakened the barrier properties of the packaging [47]. However, the water activity remained relatively stable throughout storage, ranging from 0.56 to 0.66. The water activity reflects water availability rather than the total water content. Therefore, increases in the moisture content do not necessarily result in proportional increases in the water activity because water may remain associated with proteins and other matrix components [48]. In addition, the consistently low water activity (<0.70) may help explain the absence of detectable microbial growth during storage because such conditions are generally unfavorable for the proliferation of most spoilage-associated microorganisms and foodborne pathogens [49].

**Figure 3:**
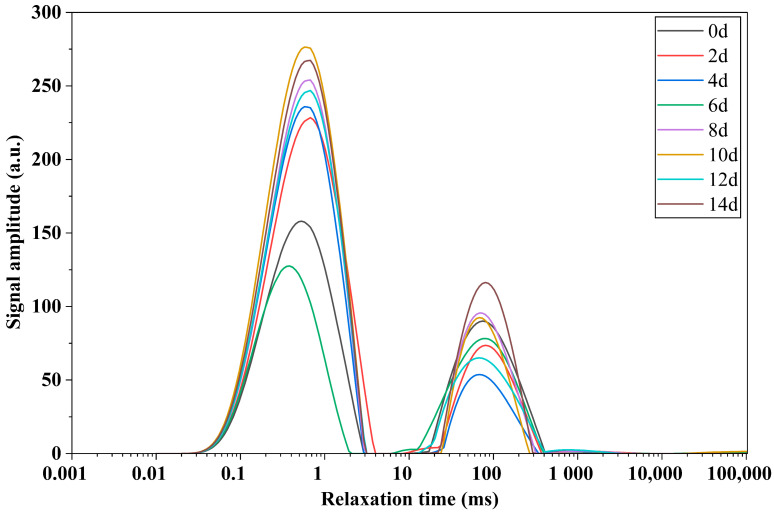
Changes in water distribution in pork jerky during storage.

The classification of water populations based on T_2_ relaxation times reflects differences in water mobility and interactions with macromolecules, where shorter T_2_ values indicate more bound water and longer T_2_ values represent more mobile water [50]. LF-NMR (Table 3 and Figure 3) identified two main relaxation peaks during storage, T_21_ and T_22_, corresponding to water with a stronger binding capacity and weaker binding capacity, respectively [51]. At all sampling points, T_21_ was the dominant peak, indicating that the water in pork jerky was mainly present as immobilized water, which is consistent with the characteristic water state of dried meat products, where water is largely restricted within the protein network [51,52]. According to the changes in the peak area proportions, the proportion of T_21_ increased during the first 4 days of storage, whereas that of T_22_ decreased, suggesting that part of the free water was converted into immobilized water during the early stages of storage, leading to reduced water mobility and enhanced water binding within the protein matrix [52,53]. On day 6, the total moisture content changed only slightly but the second relaxation peak, T_22_, increased markedly, indicating an increase in the proportion of free water and weakening of the water-binding capacity within the sample, suggesting that neither the moisture content nor the water activity alone fully reflected the alterations in the internal water state of pork jerky. The transient increase in free water on day 6 may indicate a transition stage between early storage and accelerated deterioration. Protein denaturation and structural rearrangement may have temporarily increased the water mobility, and this change was probably associated with internal physicochemical transformations [54,55]. This behavior was most likely linked with the accumulation of oxidative damage to myofibrillar proteins under prolonged high-temperature and high-humidity conditions, which gradually weakened protein–water interactions and destabilized the three-dimensional muscle protein network [56]. After reaching a threshold level of structural disruption, immobilized water confined within the protein matrix was partially released, resulting in a sharp decrease in T_21_ and a concomitant increase in T_22_. Subsequently, the system tended to reach a new equilibrium state as protein aggregation and structural reorganization proceeded, leading to partial recovery and stabilization of the water distribution. As the storage time extended, the proportion of immobilized water increased, whereas that of free water decreased. On day 14, the proportions of immobilized and free water were 74.93% and 24.84%, respectively. However, this does not necessarily indicate an improvement in the water-holding capacity, but instead it may be attributed to further protein denaturation and aggregation, as well as progressive tissue densification during prolonged storage. Consequently, some water may have become physically confined within the contracted protein network and microstructural pores, leading to reduced water mobility.

#### 3.2.5. Changes in Protein and Fat Contents

As shown in Table 2, the protein and fat contents of pork jerky remained relatively stable during storage at 65 °C and 85% RH, with no significant differences among storage times. However, this stable proximate composition does not necessarily indicate nutritional stability. Oxidative reactions during storage may impair the lipid quality, protein availability, and related functional properties, thereby reducing the nutritional value of a product [3,57].

#### 3.2.6. Changes in pH

The pH is an important indicator for evaluating the freshness, eating quality, and stability in storage of meat products [58]. As shown in Table 2, the pH of pork jerky generally tended to decrease with prolonged storage from 6.20 on day 0 to 5.80 on day 14, indicating that the acid–base balance changed significantly during storage. Previous studies found that a decrease in the pH of dried meat products during storage was generally associated with the accumulation of acidic substances and progression of various physicochemical reactions. Chmiel et al. observed that the pH of sliced pork ham decreased from 5.80 to 5.50 during storage, suggesting that meat products may undergo acidification even under packaged conditions [59]. Li et al. reported that a decrease in the pH of fermented camel jerky was related to the accumulation of metabolites, such as organic acids, indicating that acid generation makes an important contribution to pH reduction in jerky-type meat systems. Furthermore, the decrease in the pH may also have been related to the Maillard reaction, which is accompanied by the formation of certain organic acids that contribute to pH reduction [60].

#### 3.2.7. Changes in Microstructural Characteristics

**Figure 4:**
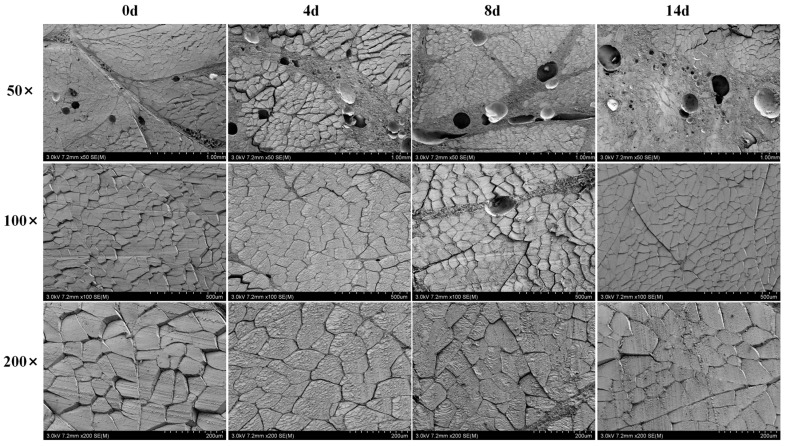
Microstructural morphology of pork jerky during storage.

Figure 4 shows the microstructural morphology of pork jerky during storage at 65 °C and 85% RH. On day 0, the muscle fibers had a relatively regular arrangement with clearly defined boundaries, and only a few small pores were observed in the tissue. As the storage period continued, the spaces between the muscle fibers gradually decreased, whereas the pores became larger. After storage for 14 days, the interfaces between muscle fiber units became blurred, with larger collapsed regions and irregular pores. Previous studies have shown that surface cracking, increased porosity, and tissue collapse in meat products during storage or processing are generally associated with water migration and matrix shrinkage [61,62]. Zhang et al. showed that myofibrillar protein oxidation can induce changes in the protein conformation, cross-linking aggregation, and structural rearrangement, leading to gradual transformation of the meat microstructure from a compact and ordered network to a rough, fractured, and irregular structure [6]. Therefore, storage under high-temperature and high-humidity conditions (65 °C and 85% RH) markedly disrupted the continuity and integrity of the internal structure of pork jerky, which may have been related to water migration, local separation of the lipid phase, and protein structural rearrangement during storage.

### 3.3. Lipid Oxidation

#### 3.3.1. Changes in PV

**Figure 5:**
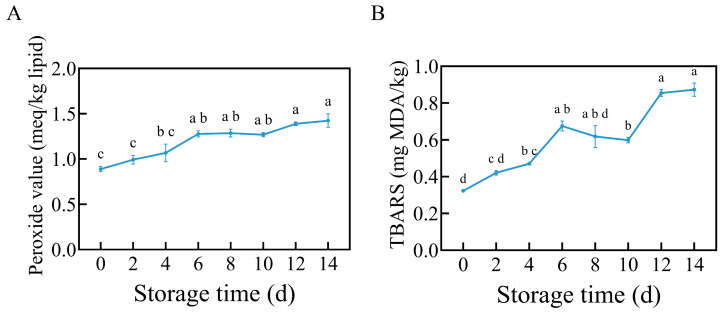
Changes in peroxide value (**A**) and TBARS value (**B**) for pork jerky during storage. Values represent the mean ± standard error (*n* = 3). Different lowercase letters indicate significant differences among storage times (*p* < 0.05).

As shown in Figure 5A, the PVs for pork jerky increased under prolonged storage, indicating that the high-temperature and high-humidity conditions induced lipid oxidation and promoted the continuous accumulation of primary lipid oxidation products in the jerky. Pork jerky is a low-moisture meat product and it was vacuum-packaged in high-barrier aluminum foil pouches with a low oxygen transmission rate and water vapor transmission rate, but lipid oxidation still occurred during storage, and the observed oxidative deterioration was unlikely to have been caused primarily by inadequate package barrier performance. Instead, the oxygen entrapped during packaging, oxygen dissolved within the product matrix, and endogenous pro-oxidant components in the raw material, such as metal ions and heme iron, may have been sufficient to drive free-radical chain reactions. Consequently, unsaturated fatty acids could have continuously generated hydroperoxides under the combined effects of heat and oxygen exposure [4,63]. It should be noted that lipid hydroperoxides are unstable intermediates that can further decompose into secondary oxidation products, including aldehydes, ketones, and alcohols, during the later stages of oxidation. These products contribute to flavor deterioration, color changes, and loss of nutritional quality, and were indicated by the concurrent increase in TBARS observed during storage. Therefore, an increase in the PV generally indicates an increased risk of subsequent oxidative damage [4,64]. Zhu et al. showed that an increase in the PV for air-dried beef in storage was accompanied by a decrease in lipid unsaturation and enhanced flavor deterioration, which are consistent with the results obtained in the present study [65].

#### 3.3.2. Changes in TBARS Values

As shown in Figure 5B, the TBARS values for pork jerky generally tended to increase during storage from 0.32 mg MDA kg^−1^ on day 0 to 0.87 mg MDA kg^−1^ on day 14. The increase was relatively rapid during the early stage of storage (0–6 days), more moderate in the middle stage (6–10 days), and highly marked in the later stage (10–14 days), indicating the continuous accumulation of secondary lipid oxidation products. Previous studies have suggested that TBARS values in the range of 0.5–1.0 mg MDA kg^−1^ may be associated with the onset of perceptible oxidative off-flavors in meat products [66]. Therefore, the final TBARS value of 0.87 mg MDA kg^−1^ in the present study approached this sensory threshold and may have contributed to the decline in sensory acceptability observed during storage. The increase in PV indicated the accumulation of primary oxidation products, whereas the continuous increase in the TBARS value suggests that lipid hydroperoxides formed in the early stage were further decomposed into secondary products, including aldehydes and ketones. Thus, PV and TBARS reflect different stages of lipid oxidation and demonstrate the progression of oxidative deterioration. Similarly, Zhao et al. observed an increase in the TBARS value during the storage of fermented pork jerky and suggested that lipid oxidation was an important contributor to shelf-life quality deterioration [67]. Aung et al. also showed that TBARS was a critical indicator of oxidative deterioration in jerky-type products, and an increase in TBARS was closely associated with intensified lipid oxidation [28]. Therefore, considering the increase in PV, color darkening, and decline in sensory quality observed in the present study, lipid oxidation can be regarded as a major contributor to quality deterioration in pork jerky. Moreover, the accumulation of aldehydes may have further promoted off-flavor formation and intensified the co-oxidation reactions with proteins.

### 3.4. Protein Oxidation

#### 3.4.1. Changes in Myoglobin Forms

**Figure 6:**
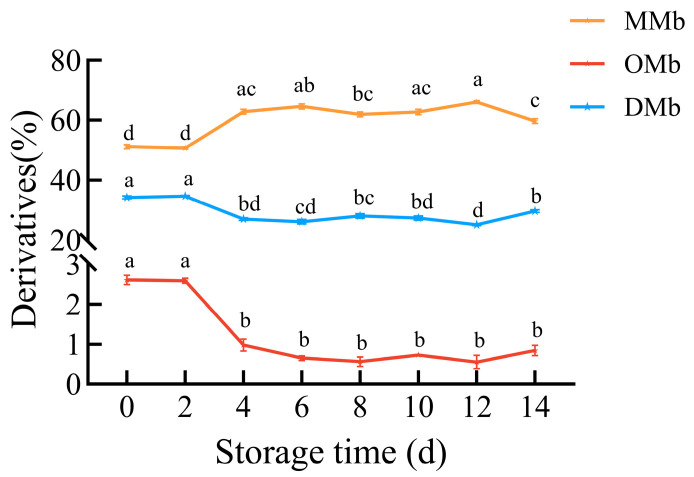
Changes in the contents of different myoglobin forms in pork jerky during storage. Values represent the mean ± standard error (*n* = 3). Different lowercase letters indicate significant differences among storage times (*p* < 0.05).

Myoglobin is a heme-containing protein where the central iron ion mainly exists in either the ferrous (Fe^2+^) or ferric (Fe^3+^) state, and the redox status of this iron is a key determinant of meat color [31]. The oxidation of myoglobin promotes the conversion of OMb and DMb into MMb, causing meat products to gradually shift from a bright red color to a grayish-brown or dark brown appearance [68]. The pork jerky was a commercially processed product that had undergone curing, thermal processing, drying, and vacuum packaging before the storage experiment. Thermal processing and exposure to oxygen during manufacturing can promote the oxidation of myoglobin, resulting in the accumulation of MMb prior to storage. Consequently, MMb was already the dominant myoglobin form on day 0 [69]. As shown in Figure 6, the initial contents of OMb, MMb, and DMb in pork jerky were 2.61%, 51.18%, and 34.23%, respectively. During storage at 65 °C and 85% RH, the OMb and DMb contents decreased, whereas the MMb content increased. These changes were particularly pronounced during the early stages of storage (0–4 days), followed by more gradual variation in the later stages. Thus, the redox forms of myoglobin in pork jerky changed during storage, where ferrous myoglobin was progressively converted into the ferric oxidized form, especially during the early storage period. Previous studies have shown that OMb is an important pigment responsible for the bright red color of meat products, and its decrease usually indicates reduced color stability [68]. A decline in DMb suggests the gradual depletion of reduced pigment forms and a decrease in the reducing capacity of the system [70], whereas MMb accumulation is closely associated with a transition of the meat color from bright red to grayish-brown or dark brown [70,71]. The results suggested that the oxidation of myoglobin progressively intensified during storage, and the pigment gradually shifted from a color-stable state to an oxidized state that was not favorable for color retention. Changes in the redox forms of myoglobin are closely related to the a* value, total color difference, and visual acceptability of meat products. Vacuum packaging can delay oxidation but it cannot completely prevent MMb formation during storage [71,72]. In addition, Zhang et al. reported a mutually reinforcing cycle between lipid and myoglobin oxidation [31]. Therefore, in addition to the color, browning degree, and lipid oxidation results observed in the present study, the conversion of OMb and DMb to MMb may be considered a key mechanism associated with color deterioration in pork jerky. This process may have been further intensified by the synergistic effect of lipid oxidation in storage.

#### 3.4.2. Changes in TVB-N

**Figure 7:**
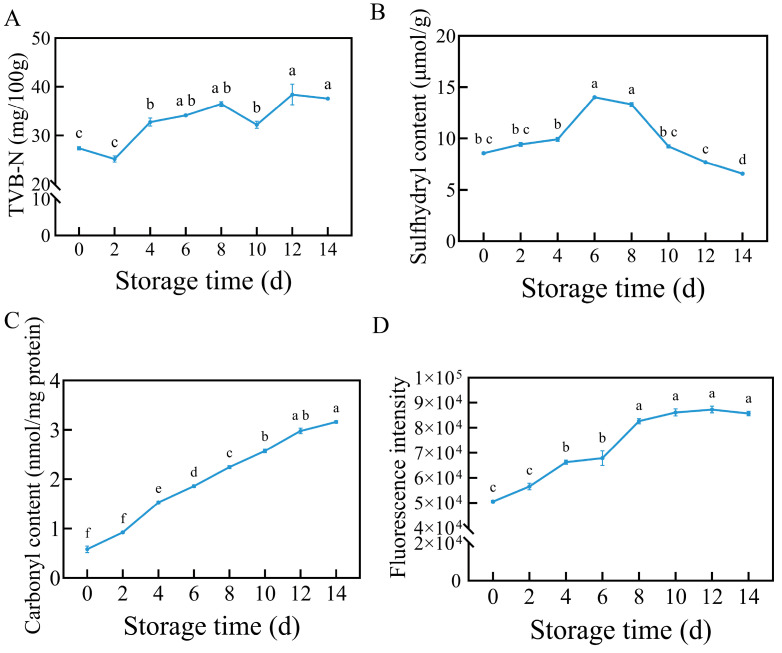
Changes in TVB-N (**A**), total sulfhydryl (**B**), carbonyl (**C**), and Schiff base contents (**D**) of pork jerky during storage. Values represent the mean ± standard error (*n* = 3). Different lowercase letters indicate significant differences among storage times (*p* < 0.05).

As shown in Figure 7A, the TVB-N content of pork jerky increased over time during storage from 27.39 mg 100 g^−1^ on day 0 to 37.60 mg 100 g^−1^ on day 14, indicating the continuous accumulation of volatile alkaline nitrogenous compounds. During the storage of meat products, endogenous enzymatic activity, protein degradation, and microbial metabolism can all contribute to an increase in TVB-N. However, in low-moisture meat products, even when microbial proliferation is limited, protein degradation, amino acid deamination, and oxidation-related reactions under high-temperature conditions may still promote the formation of volatile basic compounds [73,74]. In the present study, TVC remained below the detection limit, whereas TVB-N continuously increased, suggesting that this change was more likely to have been associated with heat-induced non-microbial protein degradation and chemical deterioration reactions, and similar results were reported by Zhao et al. [67]. In addition, Jin et al. reported that vacuum packaging could delay TVB-N formation but not completely prevent the accumulation of volatile nitrogenous compounds during storage [75]. Therefore, the increase in TVB-N observed under accelerated storage conditions primarily reflected heat-accelerated chemical deterioration, which may not be fully representative of the changes that occur under practical storage conditions.

#### 3.4.3. Changes in Total Sulfhydryl Content

The total sulfhydryl content is commonly used as an indicator of protein oxidation in meat products [45,76]. As shown in Figure 7B, the total sulfhydryl content of pork jerky increased initially and then decreased during storage, reaching a maximum on day 6, followed by a continuous decline. The increase in the total sulfhydryl content during the early stage of storage suggests that proteins may have undergone conformational loosening or partial unfolding under high-temperature and high-humidity conditions, resulting in the exposure of sulfhydryl groups that were originally buried within the protein molecules. Similar phenomena have also been reported during thermal treatment or moderate structural rearrangement of myofibrillar proteins [77,78]. The subsequent marked decrease in the total sulfhydryl content indicates that the exposed sulfhydryl groups were further involved in oxidative reactions and converted into disulfide bonds, accompanied by enhanced protein cross-linking, suggesting that protein oxidation gradually shifted from a structural unfolding stage to an aggregation and cross-linking stage [45,76]. In addition, previous studies showed that the continuous loss of protein sulfhydryl groups during storage was often accompanied by increased carbonyl formation, intensified lipid oxidation, and deterioration of functional properties. Antioxidant treatments have been reported to effectively retard sulfhydryl loss, further confirming the high sensitivity of the sulfhydryl content as an indicator of oxidative damage [79,80]. Therefore, in addition to the changes in TBARS, PV, and texture observed in the present study, storage at 65 °C and 85% RH may have markedly promoted lipid–protein co-oxidation as well as protein cross-linking and aggregation in pork jerky.

#### 3.4.4. Changes in Carbonyl Content

The protein carbonyl content is widely used as a marker of protein oxidation, and its increase generally indicates the oxidative modification of proteins and deterioration of their structure and functionality [81,82]. As shown in Figure 7C, the carbonyl content of pork jerky increased continuously during storage from 0.58 nmol mg^−1^ protein on day 0 to 3.16 nmol mg^−1^ protein on day 14, indicating that protein oxidation progressively intensified during storage. Under high-temperature and high-humidity conditions, lipid oxidation and protein oxidation often occur simultaneously. Reactive intermediates generated from lipid oxidation, such as aldehydes and ketones, can further attack proteins and promote carbonyl accumulation. Therefore, the continuous increase in the carbonyl content in the present study suggests that pork jerky underwent direct protein oxidation but also pronounced lipid–protein co-oxidation during storage [63,83]. In addition, protein oxidation induced by thermal treatment or storage can lead to the unfolding, aggregation, and functional deterioration of myofibrillar proteins. Niu et al. showed that the carbonyl content of duck myofibrillar proteins increased significantly during thermal treatment, accompanied by a decrease in the sulfhydryl content and changes in structural stability, indicating that carbonyl formation is closely associated with protein structural damage [84]. Enhanced carbonylation reflects protein oxidation itself but may also further affect the texture, water-holding capacity, and nutritional quality. Previous studies have shown that moderate to severe protein oxidation can promote subsequent browning-related reactions through protein aggregation and oxidative modification, as well as reduce the accessibility of reactive sites. These findings suggest that carbonyl accumulation may serve as an important link between protein oxidation and quality deterioration [85].

#### 3.4.5. Changes in Schiff Base Content

Schiff bases are generally formed through reactions between free amino groups in proteins and reactive carbonyl compounds. In pork jerky, Schiff bases may be formed via two distinct pathways: lipid oxidation-derived carbonyl–amine condensation and the classical Maillard reaction. Therefore, they are commonly treated as auxiliary indicators for evaluating carbonyl–amine condensation reactions, protein oxidation, and lipid–protein co-oxidation in meat products [86]. As shown in Figure 7D, the Schiff base content of pork jerky generally increased during storage, indicating that carbonyl–amine condensation reactions were continuously enhanced. First, under high-temperature and high-humidity conditions, reactive carbonyl compounds generated from lipid oxidation, such as aldehydes and ketones, can further react with amino groups in proteins, thereby promoting Schiff base formation and protein structural modification [87,88]. In addition, Schiff bases are early intermediates in the Maillard reaction. However, the Maillard reaction is a complex and dynamic process, so the Schiff base content alone cannot be used as a specific indicator to confirm progression of the Maillard reaction, although when considered together with the changes in the browning degree and total sugar content, the increase in the Schiff base content supports the occurrence of non-enzymatic browning. Overall, these results indicate that reactions between carbonyl compounds and protein amino groups were markedly promoted in pork jerky during storage, reflecting intensified oxidation but also suggesting a clear interaction between oxidative reactions and non-enzymatic browning, which may have jointly contributed to changes in the color, texture, and nutritional quality [86].

### 3.5. Maillard Reaction

#### 3.5.1. Changes in Total Sugar Content

**Figure 8:**
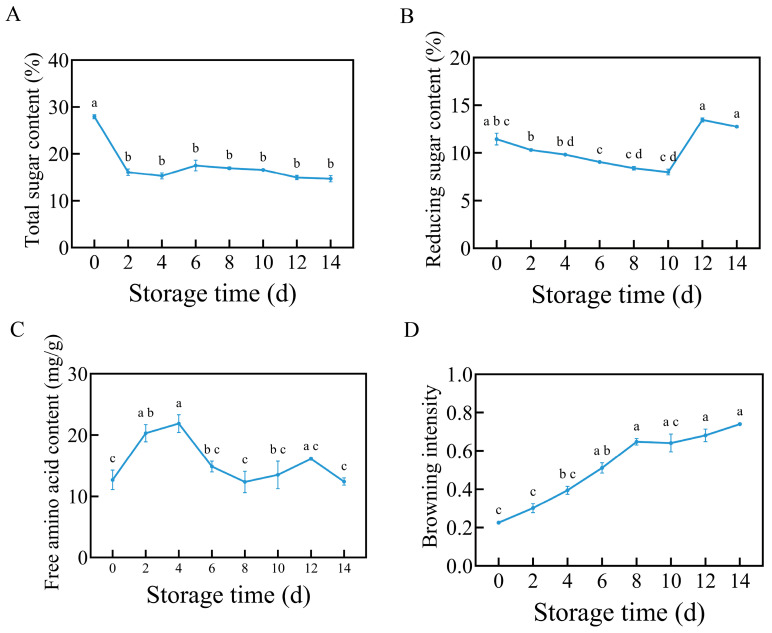
Changes in (**A**) total sugar content, (**B**) reducing sugar content, (**C**) free amino acid content, and (**D**) browning degree for pork jerky during storage. Values represented the mean ± standard error (*n* = 3). Different lowercase letters indicate significant differences among storage times (*p* < 0.05).

The total sugar content reflects sugar consumption during thermal degradation and browning reactions [9,89]. As shown in Figure 8A, the total sugar content of pork jerky generally decreased under storage in conditions of 65 °C and 85% RH, where it decreased rapidly from 27.94% on day 0 to ~16.09% on day 2 and then declined slowly to 14.68% on day 14, indicating that high-temperature and high-humidity storage markedly promoted the early depletion of measurable total sugars. The sharp decrease in the total sugar content during the initial stage of storage may have been associated with sucrose transformation and its involvement in non-enzymatic browning reactions. Previous studies have shown that sucrose can undergo cleavage or conversion under heating conditions to generate intermediate products that participate in subsequent reactions, thereby reducing the levels of measurable sugars [90,91]. Therefore, the continuous decrease in the total sugar content in the present study suggests that the degradation or consumption of sugar occurred in pork jerky, even under vacuum packaging in aluminum foil pouches. This process may have been important for browning accumulation and quality deterioration.

#### 3.5.2. Changes in Reducing Sugar Content

The reducing sugar content reflects the consumption of key substrates involved in the Maillard reaction during storage and processing [9,92,93]. As shown in Figure 8B, the reducing sugar content of pork jerky decreased initially and then increased during storage, where it gradually decreased from 11.45% on day 0 to 8.00% on day 10, before rapidly increasing to 13.47% on day 12, followed by a slight decline to 12.76% on day 14, which was higher than the initial level. The early decrease in the reducing sugar content suggests the continuous consumption of reducing sugars, possibly due to their participation as carbonyl donors in the Maillard reaction, as also shown in the studies by Liu et al. and Bolchini et al. [94,95]. Previous studies have demonstrated that reducing sugars can react with amino compounds even under mild processing or storage conditions, thereby affecting color and flavor formation in meat products [89,96]. By contrast, the increase in the reducing sugar content during the later stage of storage may have been related to the hydrolysis of sucrose into glucose and fructose. It has been reported that sucrose can be converted into reducing sugars during thermal processing and subsequently participate in browning reactions [97]. Therefore, under high-temperature conditions, the contents of reducing sugars in pork jerky may exhibit a dynamic pattern characterized by early-stage consumption and late-stage replenishment. However, the mechanism responsible for this increase remains unclear, and thus this explanation remains tentative and requires further verification.

#### 3.5.3. Changes in Free Amino Acid Content

Free amino acids are mainly derived from muscle protein degradation and serve as important precursors for flavor formation in meat products. They are also key substrates involved in the Maillard reaction and Strecker degradation [96,98]. As shown in Figure 8C, the free amino acid content of pork jerky stored at 65 °C and 85% RH increased initially and then decreased, rising from 12.70% on day 0 to 21.88% on day 4, followed by a decline to 12.42% on day 14, indicating that protein degradation occurred to some extent during the early stage of storage to promote the release of free amino acids, whereas the subsequent decrease suggests their further involvement in downstream reactions. Kathuria et al. reported that the Maillard reaction is essentially a non-enzymatic reaction between reducing sugars and free amino groups that consumes amino compounds and affects food color and flavor development [99]. In addition, Wei et al. found that glucose and free amino acids were gradually depleted during the roasting of lamb, accompanied by the increased formation of volatile flavor compounds, suggesting that free amino acids can be continuously converted into flavor- and browning-related products during thermal processing and storage. Therefore, the early increase in free amino acid content in the present study may have been associated with protein hydrolysis and the further degradation of small peptides under high-temperature conditions, whereas the later decrease may have been due to their continuous consumption in subsequent reactions [100].

#### 3.5.4. Changes in Browning Degree

The absorbance at 420 nm (A420) is commonly used to characterize the formation of melanoidin pigments produced during the advanced stages of non-enzymatic browning reactions, and thus it is considered an indicator of the accumulation of brown high-molecular-weight products during storage. However, A420 reflects the overall formation of colored compounds rather than specific Maillard reaction intermediates or end products. This measurement provides a direct indication of the color deterioration associated with non-enzymatic browning and its possible synergistic interaction with lipid oxidation during storage or processing [101,102]. As shown in Figure 8D, the degree of browning for pork jerky increased continuously under storage conditions of 65 °C and 85% RH from 0.22 on day 0 to 0.74 on day 14. The increase was more rapid during the first 8 days of storage and then tended to become more gradual in the later stage, indicating that high-temperature and high-humidity storage markedly promoted non-enzymatic browning. Browning is mainly associated with the Maillard reaction, where reducing sugars react with free amino groups through amino–carbonyl reactions to progressively form brown high-molecular-weight products. However, the carbonyl compounds generated from oxidation may also participate in carbonyl–amine condensation reactions and contribute to browning development under storage conditions [99]. Fan et al. showed that lipid oxidation can act synergistically with non-enzymatic browning during storage to promote color darkening because the carbonyl compounds generated from oxidation may further participate in browning reactions and accelerate overall color deterioration [101]. Therefore, the continuous increase in the browning degree indicated the progressive accumulation of browning products in pork jerky during storage. These reactions were closely associated with the observed color darkening and decline in sensory acceptability [92].

### 3.6. Changes in Protein Digestive Characteristics

**Figure 9:**
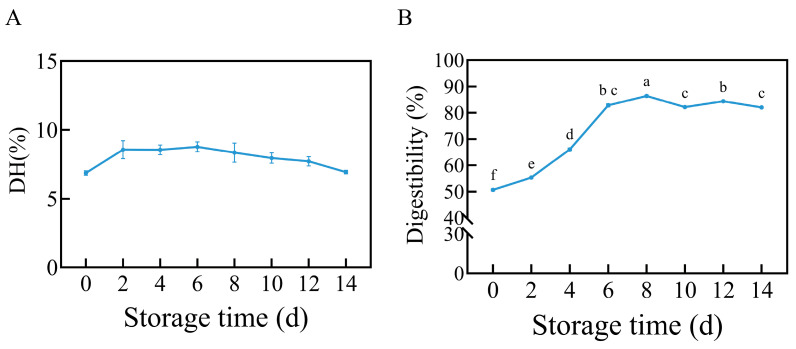
Changes in DH (**A**) and protein digestibility (**B**) for pork jerky during storage. Values represented the mean ± standard error (*n* = 3). Different lowercase letters indicate significant differences among storage times (*p* < 0.05).

Protein digestibility and DH are important indicators of protein nutritional quality and digestibility. As shown in Figure 9, the in vitro digestibility of pork jerky increased significantly in the early stage of storage but tended to stabilize in the later stage of storage, whereas DH only fluctuated slightly within the range of 6.87–8.77%, with no significant differences among time points (*p* > 0.05). Thus, storage under high-temperature and high-humidity conditions markedly improved the in vitro digestibility of pork jerky proteins but had limited effects on the final extent of protein hydrolysis. The rapid increase in digestibility during the early stage of storage may have been due to heat-induced protein denaturation and partial unfolding exposing previously buried enzymatic cleavage sites and facilitating the interaction between digestive enzymes and protein substrates [103,104]. As the storage period continued, the increase in the carbonyl content and Schiff base formation, as well as the decrease in sulfhydryl groups, indicated enhanced protein oxidation and non-enzymatic browning reactions. Excessive oxidation can induce disulfide bond formation, cross-linking aggregation, and conformational rearrangement, reducing protein flexibility and limiting enzyme accessibility [103,105,106]. In addition, Schiff base formation and subsequent Maillard-type reactions may block reactive amino groups, particularly lysine residues, and promote the formation of digestion-resistant structures to negatively affect protein digestibility [107]. Therefore, the plateau in digestibility observed during the later stages of storage may be attributed to a balance between the digestion-promoting effects of protein unfolding and the digestion-limiting effects associated with oxidation-induced aggregation and non-enzymatic browning reactions. By contrast, DH did not change significantly throughout storage, indicating that storage did not markedly alter the overall degree of peptide bond cleavage at the end point of in vitro digestion. These results suggest that the structural modifications induced during storage mainly affected enzyme accessibility rather than the final extent of protein hydrolysis.

### 3.7. Multivariate Statistical Analysis

**Figure 10:**
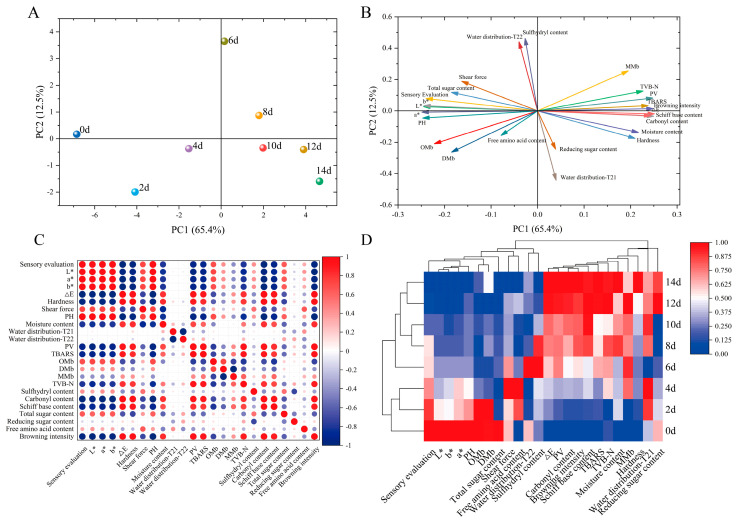
Multivariate analysis of quality deterioration patterns in vacuum-packaged pork jerky during accelerated storage. (**A**) Principal component analysis score plot, (**B**) principal component analysis loading plot, (**C**) Spearman’s correlation coefficients, and (**D**) hierarchical cluster analysis heatmap.

Principal component analysis, Spearman’s correlation coefficients, and hierarchical cluster analysis were employed to assess all of the measured indicators to comprehensively clarify the quality deterioration characteristics of pork jerky during storage. The results obtained by principal component analysis showed that PC1 and PC2 explained 65.4% and 12.5% of the total variance, respectively, with a cumulative explanatory rate of 77.9%, indicating that the first two principal components effectively reflected the overall changes in pork jerky quality during storage. As shown in Figure 10A, as the storage period continued, the samples gradually shifted from the negative side to the positive side along PC1, and the samples from day 14 were farthest from those on day 0, indicating that quality deterioration continuously intensified. The loading plot (Figure 10B) showed that the sensory score, L*, a*, b*, OMb, DMb, and total sugar content values were mainly distributed in the negative region of PC1, whereas the ΔE, hardness, PV, TBARS, TVB-N, MMb, carbonyl content, Schiff base content, and browning degree values were mainly distributed in the positive region of PC1. In particular, the PV, TBARS, carbonyl content, Schiff base content, MMb, and browning degree values had relatively high positive loadings on PC1, indicating that lipid oxidation, protein oxidation, myoglobin oxidation, and oxidation-related browning were the main factors associated with sample separation and quality deterioration. Therefore, PC1 can be regarded as an oxidation-driven deterioration axis.

The Spearman’s correlation coefficients further verified these findings (Figure 10C). ΔE was significantly positively correlated with the carbonyl content (r = 1.00), browning degree (r = 0.98), PV (r = 0.93), Schiff base content (r = 0.93), TBARS (r = 0.90), and TVB-N (r = 0.81), but negatively correlated with the OMb (r = −0.69), DMb (r = −0.43), and color parameters. In particular, the correlations for ΔE with the browning degree, carbonyl content, and lipid oxidation indicators were markedly higher than that with MMb (r = 0.43), indicating that the overall color deterioration by pork jerky during storage was not due only to myoglobin oxidation but was more strongly related to oxidation product-mediated browning reactions. Therefore, oxidation-related browning may have been important for color deterioration under the high-temperature and high-humidity conditions. In addition, the hardness had strong positive correlations with the Schiff base content (r = 0.90), carbonyl content (r = 0.88), PV (r = 0.88), and TBARS (r = 0.83), but weak correlations with the water distribution parameters T_21_ and T_22_ (|r| ≤ 0.14). Thus, texture hardening was mainly caused by the protein cross-linking and structural densification induced by protein oxidation and lipid oxidation, rather than by water migration.

The hierarchical cluster analysis results (Figure 10D) showed that the samples from days 0–4 clustered together, representing the early storage stage with relatively stable quality. The samples from days 8–14 clustered into another group, representing the stage of rapid quality decline, while the sample from day 6 was located between the two clusters and may correspond to a key transition point from slow quality change to rapid deterioration. The clustering of indicators further showed that oxidation-related indicators, including the PV, TBARS, carbonyl content, Schiff base content, MMb, and browning degree, grouped in the same branch, whereas the sensory score, color parameters, and ferrous myoglobin-related indicators clustered in another branch. This clustering pattern indicates that lipid oxidation, protein oxidation, myoglobin oxidation, and oxidation-related browning did not occur independently but instead together formed an interconnected oxidative deterioration network. These results provide strong statistical evidence to support the oxidation-dominated deterioration mechanism proposed in this study.

## 4. Conclusions

This study systematically evaluated the quality deterioration process for high-barrier vacuum-packaged pork jerky during storage under high-temperature and high-humidity conditions (65 °C and 85% RH), and comprehensively investigated the underlying mechanisms responsible for deterioration. The results showed that the product reached the shelf-life endpoint on day 14, where deterioration was mainly characterized by color darkening, texture hardening, microstructural collapse, and redistribution of water populations. The microbial counts remained below the detection limit throughout storage, confirming that chemical deterioration was the dominant process. Lipid oxidation, protein oxidation, and Maillard-type reactions occurred simultaneously. In particular, water migration and rearrangement of the protein network were the key microstructural causes of texture hardening. The multivariate analysis suggested that oxidative processes were the predominant drivers of quality deterioration under this condition. These findings indicate that even under high-barrier vacuum packaging, high-temperature and high-humidity conditions can accelerate oxidative cross-linking and non-enzymatic browning, challenging the conventional assumption that packaging alone can effectively prevent deterioration. This study provides a theoretical basis for pork jerky shelf-life assessment and quality control under extreme storage conditions.

## Figures and Tables

**Table 1:** Sensory evaluation scores for pork jerky during storage.

Storage Time	Overall Acceptability	Rejection Rate (%)
0 d	7.90 ± 0.35 ^a^	0
2 d	7.30 ± 0.21 ^a^	0
4 d	6.40 ± 0.52 ^ab^	0
6 d	5.60 ± 0.40 ^bc^	0
8 d	5.40 ± 0.45 ^bc^	10
10 d	3.60 ± 0.40 ^cd^	20
12 d	2.90 ± 0.62 ^cd^	40
14 d	2.40 ± 0.31 ^d^	50

Values represent the mean ± standard error. Different letters within the same column indicate significant differences (*p* < 0.05).

**Table 2:** Physicochemical properties of pork jerky during storage.

Storage Time	TVC (CFU/g)	Yeast (CFU/g)	Mold (CFU/g)	*Bacillus cereus* (CFU/g)	Protein Content (g 100 g^−1^)	Fat Content (g 100 g^−1^)	pH
0 d	<10.00	<10.00	<10.00	<10.00	37.23 ± 1.05 ^a^	10.23 ± 0.44 ^a^	6.21 ± 0.04 ^ab^
2 d	<10.00	<10.00	<10.00	<10.00	35.83 ± 1.28 ^a^	11.03 ± 0.66 ^a^	6.09 ± 0.02 ^ab^
4 d	<10.00	<10.00	<10.00	<10.00	34.77 ± 0.37 ^a^	10.43 ± 0.90 ^a^	6.01 ± 0.02 ^a^
6 d	<10.00	<10.00	<10.00	<10.00	36.37 ± 1.57 ^a^	11.23 ± 0.32 ^a^	5.92 ± 0.02 ^ab^
8 d	<10.00	<10.00	<10.00	<10.00	35.33 ± 1.35 ^a^	9.67 ± 0.13 ^a^	5.91 ± 0.03 ^ab^
10 d	<10.00	<10.00	<10.00	<10.00	34.03 ± 0.12 ^a^	10.33 ± 0.22 ^a^	5.91 ± 0.01 ^ab^
12 d	<10.00	<10.00	<10.00	<10.00	38.40 ± 2.11 ^a^	10.27 ± 0.28 ^a^	5.89 ± 0.00 ^a^
14 d	<10.00	<10.00	<10.00	<10.00	35.97 ± 0.78 ^a^	10.67 ± 0.43 ^a^	5.80 ± 0.01 ^b^

Values represent the mean ± standard error. Different letters within the same column indicate significant differences (*p* < 0.05).

**Table 3:** Changes in the proportions of different water populations, moisture content, and water activity (a_w_) for pork jerky during storage.

Storage Time	T_21_ (%)	T_22_ (%)	Moisture Content (g 100 g^−1^)	a_w_
0 d	68.95 ± 1.46 ^bc^	30.76 ± 1.40 ^ab^	11.62 ± 0.09 ^d^	0.57 ± 0.001 ^f^
2 d	79.40 ± 2.35 ^ab^	20.37 ± 2.31 ^bc^	12.53 ± 0.08 ^c^	0.62 ± 0.001 ^b^
4 d	81.55 ± 2.09 ^ab^	18.26 ± 2.11 ^bc^	12.44 ± 0.05 ^c^	0.61 ± 0.001 ^e^
6 d	61.16 ± 0.74 ^c^	38.37 ± 1.08 ^a^	12.41 ± 0.09 ^cd^	0.56 ± 0.001 ^g^
8 d	77.29 ± 0.67 ^ab^	22.44 ± 0.68 ^bc^	13.50 ± 0.17 ^ab^	0.66 ± 0.001 ^a^
10 d	79.83 ± 1.17 ^a^	19.97 ± 1.16 ^c^	13.10 ± 0.05 ^ac^	0.62 ± 0.001 ^b^
12 d	76.27 ± 1.68 ^ab^	23.46 ± 1.75 ^bc^	12.86 ± 0.40 ^bc^	0.62 ± 0.001 ^c^
14 d	74.93 ± 1.61 ^ab^	24.84 ± 1.43 ^bc^	13.73 ± 0.04 ^a^	0.61 ± 0.001 ^d^

Values represent the mean ± standard error. Different letters within the same column indicate significant differences (*p* < 0.05).

## Data Availability

The original contributions presented in the study are included in the article/Supplementary Materials; further inquiries can be directed to the corresponding authors.

## Supplementary Materials

The following supporting information can be downloaded at: https://www.mdpi.com/article/10.3390/foods15142565/s1, Table S1. Sensory evaluation scores of pork jerky during storage.

## Abbreviations

The following abbreviations are used in this manuscript:

RH	relative humidity
OPA	o-phthalaldehyde
TVCs	total viable counts
MYP	mannitol egg yolk polymyxin
LF-NMR	low-field nuclear magnetic resonance
aw	Water activity
SEM	Scanning electron microscopy
PV	peroxide value
TBARS	thiobarbituric acid reactive substances
OMb	oxymyoglobin
DMb	deoxymyoglobin
MMb	metmyoglobin
TVB-N	total volatile basic nitrogen
DTNB	5,5′-Dithiobis(2-nitrobenzoic acid)
DNS	3,5-dinitrosalicylic acid
TCA	trichloroacetic acid
DH	degree of hydrolysis
ISO	International Organization for Standardization
A420	absorbance at 420 nm
