# Supplementary material for: Mechanisms Associated with Quality Deterioration for Vacuum-Packaged Pork Jerky During Accelerated High-Temperature and High-Humidity Storage
Source: Foods. 2026 Jul 21;15(14):2565. doi: 10.3390/foods15142565 (PMC13409683; doi:10.3390/foods15142565)
Supplement: Supplementary file 1 [file foods-15-02565-s001.zip › foods-4352508-supplementary.pdf]

Table S1. Sensory evaluation scores of pork jerky during storage.

Storage time	Color	Appearance	Aroma	Taste	Texture
0d	8.10 ± 0.28^{a}	8.40 ± 0.31^{a}	8.00 ± 0.39^{a}	8.20 ± 0.25^{a}	6.90 ± 0.48^{a}
2d	8.00 ± 0.26^{a}	8.33 ± 0.33^{a}	8.33 ± 0.21^{a}	8.33 ± 0.33^{a}	7.17 ± 0.31^{a}
4d	6.90 ± 0.43^{ab}	8.30 ± 0.26^{a}	6.70 ± 0.47^{ab}	7.10 ± 0.38^{a}	5.90 ± 0.86^{ab}
6d	5.70 ± 0.42^{b}	7.60 ± 0.37^{a}	6.60 ± 0.56^{ab}	6.60 ± 0.69^{ab}	5.40 ± 0.56^{abc}
8d	5.30 ± 0.52^{bc}	6.60 ± 0.50^{ab}	5.80 ± 0.66^{abc}	5.30 ± 0.80^{abc}	5.20 ± 0.55^{abc}
10d	3.60 ± 0.40^{cd}	4.90 ± 0.55^{bc}	4.20 ± 0.61^{bd}	3.50 ± 0.69^{bd}	3.50 ± 0.58^{bd}
12d	2.80 ± 0.44^{d}	5.00 ± 0.52^{bc}	3.70 ± 0.52^{cd}	2.50 ± 0.82^{cd}	3.10 ± 0.48^{cd}
14d	2.30 ± 0.26^{d}	4.30 ± 0.30^{c}	2.50 ± 0.40^{d}	1.50 ± 0.50^{d}	1.80 ± 0.47^{d}

Values are expressed as mean ± standard error. Different letters within the same column indicate significant differences ($p < 0.05$).
